# 5-*p*-Tolyl-1,2,3,3a-tetra­hydro­benzo[*e*]pyrrolo­[2,1-*b*][1,3]oxazepin-10(5*H*)-one

**DOI:** 10.1107/S1600536811020964

**Published:** 2011-06-11

**Authors:** Yun-Zhou Jin, Cai-E Liu, Rong-Hua Zhang, Da-Xu Fu, Yao-Kang Lv

**Affiliations:** a Chemistry Department, Tongji University, Shanghai 200092, People’s Republic of China

## Abstract

The structure of the title compound, C_19_H_19_NO_2_, contains a seven-membered ring, which is fused to one five- and one six-membered ring, and carries a tolyl substituent. The two benzene rings are oriented relative to each other at a dihedral angle of 86.90 (7)°. In the crystal, mol­ecules are linked by weak inter­molecular C—H⋯O hydrogen bonds.

## Related literature

For general background to asymmetric photochemical reactions, see: Gould *et al.* (2001 ▶); Grätzel (2001 ▶); Korzeniewski & Zoladz (2001 ▶); Aubert *et al.* (2000 ▶). For related structures, see: Basarić *et al.* (2008 ▶); Griesbeck *et al.* (1997 ▶, 1999 ▶, 2002 ▶).
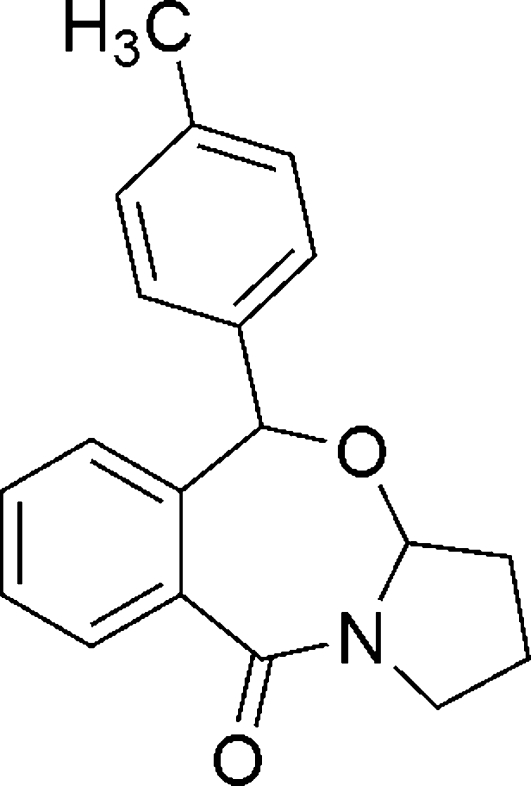


## Experimental

### 

#### Crystal data

C_19_H_19_NO_2_
*M* *_r_* = 293.35
Monoclinic, 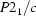

*a* = 8.237 (3) Å
*b* = 16.868 (6) Å
*c* = 11.267 (4) Å
β = 100.851 (6)°
*V* = 1537.6 (10) Å^3^
*Z* = 4
Mo *K*α radiation
μ = 0.08 mm^−1^
*T* = 296 K
0.20 × 0.18 × 0.17 mm

#### Data collection

Bruker APEXII area-detector diffractometer
Absorption correction: empirical (using intensity measurements) (*SADABS*; Sheldrick, 1996 ▶) *T*_min_ = 0.736, *T*_max_ = 1.000
13060 measured reflections
3501 independent reflections
2338 reflections with *I* > 2σ(*I*)
*R*_int_ = 0.030

#### Refinement

*R*[*F* ^2^ > 2σ(*F* ^2^)] = 0.053
*wR*(*F* ^2^) = 0.188
*S* = 1.01
3501 reflections
199 parameters
H-atom parameters constrained
Δρ_max_ = 0.46 e Å^−3^
Δρ_min_ = −0.22 e Å^−3^

### 

Data collection: *APEX2* (Bruker, 2006 ▶); cell refinement: *SAINT* (Bruker, 2006 ▶); data reduction: *SAINT*; program(s) used to solve structure: *SHELXS97* (Sheldrick, 2008 ▶); program(s) used to refine structure: *SHELXL97* (Sheldrick, 2008 ▶); molecular graphics: *DIAMOND* (Brandenburg & Putz, 2004 ▶); software used to prepare material for publication: *SHELXTL* (Sheldrick, 2008 ▶).

## Supplementary Material

Crystal structure: contains datablock(s) I, global. DOI: 10.1107/S1600536811020964/ez2247sup1.cif

Structure factors: contains datablock(s) I. DOI: 10.1107/S1600536811020964/ez2247Isup2.hkl

Supplementary material file. DOI: 10.1107/S1600536811020964/ez2247Isup3.cml

Additional supplementary materials:  crystallographic information; 3D view; checkCIF report

## Figures and Tables

**Table 1:** Hydrogen-bond geometry (Å, °)

*D*—H⋯*A*	*D*—H	H⋯*A*	*D*⋯*A*	*D*—H⋯*A*
C5—H5*B*⋯O2^i^	0.96	2.42	3.354 (3)	163
C17—H17*A*⋯O2^ii^	0.98	2.38	3.357 (3)	174

Symmetry codes: (i) 
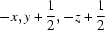
; (ii) 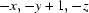.

**Fig. 1.**
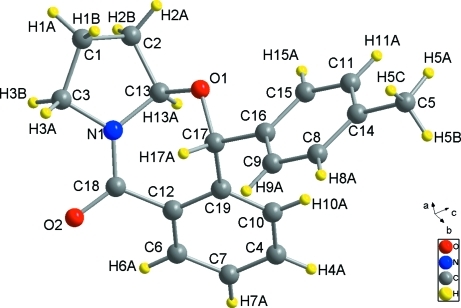
The molecular structure of the title compound showing the atomic numbering scheme.

**Fig. 2.**
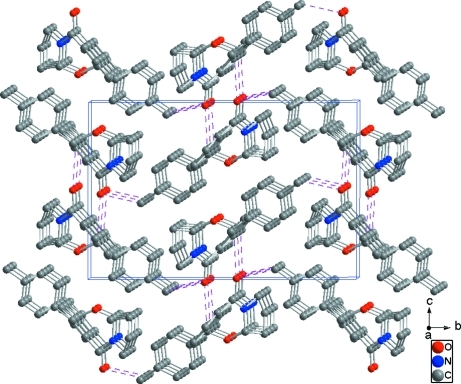
Partial packing view showing the C—H···O interactions and the formation of two-dimensional layer in the bc plane.

## supplementary crystallographic information

### Comment

Asymmetric photochemistry is one of the most important reactions in organic chemistry. Many compounds can be obtained in one step, but can not be synthesized in the ground state (Gould *et al.*,2001; Grätzel, 2001; Korzeniewski & Zoladz, 2001; Aubert *et al.*, 2000). Benzophenone acylamide derivatives can form a seven-membered ring with high stereoselectivity through intramolecular photoinduced decarboxylation and cyclization (Basarić *et al.*, 2008; Griesbeck *et al.*, 1997; Griesbeck *et al.*, 1999; Griesbeck *et al.*, 2002). We report herein the crystal structure and synthesis of the title compound.

The structure of the title compound which contains a seven-membered ring, a five-membered ring and two six-membered rings is shown in Fig. 1. The dihedral angle between the two benzene rings is 86.90 (7)°. Atoms C13 and C17 of the title compound are chiral centers. There are weak C—H···O hydrogen bonds which link neighboring molecules to form a two-dimensional layer in the *bc* plane (Fig. 2).

### Experimental

The title compound was the main product from the photoreaction of (*S*)-1-(2-(4-methylbenzoyl) benzoyl)pyrrolidine-2-carboxylic acid under N_2_ for 10 h. The compound was purified by flash column chromatography (silica gel column, petroleum ether/ethyl acetate=3/1). Colorless crystals for the X-ray crystallographic studies were gained from slow evaporation of a dichloromethane solution.

### Refinement

The hydrogen atoms attached to carbon atoms were located by geometrical calculation using a riding model, with C—H distances: phenyl C-H = 0.93 Å; primary C-H = 0.96 Å, secondary C-H = 0.97 Å; tertiary C-H: 0.98 Å [*U*_iso_(H) = 1.2*U*_eq_(C)].

### Crystal data

**Table:**

C_19_H_19_NO_2_	*F*(000) = 624
*M**_r_* = 293.35	*D*_x_ = 1.267 Mg m^−^^3^
Monoclinic, *P*2_1_/*c*	Mo *K*α radiation, λ = 0.71073 Å
Hall symbol: -P 2ybc	Cell parameters from 4317 reflections
*a* = 8.237 (3) Å	θ = 2.2–27.5°
*b* = 16.868 (6) Å	µ = 0.08 mm^−^^1^
*c* = 11.267 (4) Å	*T* = 296 K
β = 100.851 (6)°	Prism, colourless
*V* = 1537.6 (10) Å^3^	0.20 × 0.18 × 0.17 mm
*Z* = 4	

### Data collection

**Table:**

Bruker APEXII area-detector diffractometer	3501 independent reflections
Radiation source: fine-focus sealed tube	2338 reflections with *I* > 2σ(*I*)
graphite	*R*_int_ = 0.030
ω scans	θ_max_ = 27.5°, θ_min_ = 2.2°
Absorption correction: empirical (using intensity measurements) (*SADABS*; Sheldrick, 1996)	*h* = −9→10
*T*_min_ = 0.736, *T*_max_ = 1.000	*k* = −21→21
13060 measured reflections	*l* = −12→14

### Refinement

**Table:**

Refinement on *F*^2^	Primary atom site location: structure-invariant direct methods
Least-squares matrix: full	Secondary atom site location: difference Fourier map
*R*[*F*^2^ > 2σ(*F*^2^)] = 0.053	Hydrogen site location: inferred from neighbouring sites
*wR*(*F*^2^) = 0.188	H-atom parameters constrained
*S* = 1.01	*w* = 1/[σ^2^(*F*_o_^2^) + (0.105*P*)^2^] where *P* = (*F*_o_^2^ + 2*F*_c_^2^)/3
3501 reflections	(Δ/σ)_max_ < 0.001
199 parameters	Δρ_max_ = 0.46 e Å^−^^3^
0 restraints	Δρ_min_ = −0.22 e Å^−^^3^

### Special details

**Table:**

Geometry. All e.s.d.'s (except the e.s.d. in the dihedral angle between two l.s. planes) are estimated using the full covariance matrix. The cell e.s.d.'s are taken into account individually in the estimation of e.s.d.'s in distances, angles and torsion angles; correlations between e.s.d.'s in cell parameters are only used when they are defined by crystal symmetry. An approximate (isotropic) treatment of cell e.s.d.'s is used for estimating e.s.d.'s involving l.s. planes.
Refinement. Refinement of *F*^2^ against ALL reflections. The weighted *R*-factor *wR* and goodness of fit *S* are based on *F*^2^, conventional *R*-factors *R* are based on *F*, with *F* set to zero for negative *F*^2^. The threshold expression of *F*^2^ > σ(*F*^2^) is used only for calculating *R*-factors(gt) *etc*. and is not relevant to the choice of reflections for refinement. *R*-factors based on *F*^2^ are statistically about twice as large as those based on *F*, and *R*- factors based on ALL data will be even larger.

### Fractional atomic coordinates and isotropic or equivalent isotropic displacement parameters (Å^2^)

**Table:**

	*x*	*y*	*z*	*U*_iso_*/*U*_eq_	
O1	0.15809 (16)	0.47030 (7)	0.33133 (12)	0.0557 (4)	
N1	0.00869 (19)	0.40627 (9)	0.15419 (14)	0.0512 (4)	
C1	0.2534 (3)	0.33184 (14)	0.1825 (2)	0.0753 (7)	
H1A	0.2936	0.2797	0.1659	0.113*	
H1B	0.3427	0.3699	0.1866	0.113*	
O2	−0.18305 (19)	0.44457 (9)	−0.00756 (13)	0.0663 (4)	
C2	0.1855 (3)	0.33170 (12)	0.2983 (2)	0.0704 (6)	
H2A	0.2735	0.3382	0.3681	0.106*	
H2B	0.1272	0.2827	0.3069	0.106*	
C3	0.1049 (3)	0.35569 (13)	0.0862 (2)	0.0673 (6)	
H3A	0.1393	0.3849	0.0210	0.101*	
H3B	0.0417	0.3096	0.0531	0.101*	
C4	−0.3496 (3)	0.58920 (13)	0.3392 (2)	0.0637 (6)	
H4A	−0.3995	0.6193	0.3914	0.096*	
C5	0.4603 (3)	0.79714 (14)	0.5623 (2)	0.0754 (7)	
H5A	0.4984	0.7799	0.6440	0.113*	
H5B	0.3937	0.8438	0.5622	0.113*	
H5C	0.5535	0.8089	0.5255	0.113*	
C6	−0.3713 (2)	0.50009 (12)	0.17358 (19)	0.0583 (5)	
H6A	−0.4362	0.4709	0.1124	0.087*	
C7	−0.4450 (3)	0.54405 (13)	0.2524 (2)	0.0664 (6)	
H7A	−0.5592	0.5429	0.2463	0.100*	
C8	0.2897 (3)	0.74206 (12)	0.3717 (2)	0.0595 (5)	
H8A	0.3033	0.7900	0.3341	0.089*	
C9	0.2009 (2)	0.68221 (12)	0.30601 (19)	0.0558 (5)	
H9A	0.1555	0.6906	0.2250	0.084*	
C10	−0.1795 (2)	0.59058 (11)	0.35038 (18)	0.0536 (5)	
H10A	−0.1167	0.6224	0.4092	0.080*	
C11	0.3356 (3)	0.66015 (12)	0.54475 (18)	0.0589 (5)	
H11A	0.3809	0.6522	0.6259	0.088*	
C12	−0.2000 (2)	0.49935 (11)	0.18561 (16)	0.0470 (4)	
C13	0.0680 (2)	0.40196 (11)	0.28510 (17)	0.0529 (5)	
H13A	−0.0237	0.3922	0.3274	0.079*	
C14	0.3585 (2)	0.73232 (11)	0.49200 (19)	0.0545 (5)	
C15	0.2468 (3)	0.59949 (12)	0.47968 (18)	0.0578 (5)	
H15A	0.2331	0.5516	0.5175	0.087*	
C16	0.1782 (2)	0.60969 (11)	0.35837 (16)	0.0463 (4)	
C17	0.0854 (2)	0.54463 (10)	0.28326 (16)	0.0463 (4)	
H17A	0.1067	0.5501	0.2010	0.069*	
C18	−0.1249 (2)	0.44804 (11)	0.10158 (17)	0.0505 (5)	
C19	−0.1014 (2)	0.54521 (10)	0.27503 (16)	0.0442 (4)	

### Atomic displacement parameters (Å^2^)

**Table:**

	*U*^11^	*U*^22^	*U*^33^	*U*^12^	*U*^13^	*U*^23^
O1	0.0487 (8)	0.0543 (8)	0.0601 (9)	0.0093 (6)	0.0006 (6)	−0.0076 (6)
N1	0.0479 (9)	0.0542 (8)	0.0526 (9)	0.0007 (7)	0.0119 (7)	−0.0068 (7)
C1	0.0647 (14)	0.0654 (13)	0.0979 (19)	0.0143 (11)	0.0205 (13)	−0.0125 (12)
O2	0.0627 (9)	0.0857 (11)	0.0481 (8)	−0.0147 (8)	0.0046 (7)	−0.0054 (7)
C2	0.0688 (14)	0.0576 (12)	0.0835 (16)	0.0105 (10)	0.0109 (12)	−0.0003 (11)
C3	0.0717 (14)	0.0625 (12)	0.0727 (15)	−0.0003 (11)	0.0265 (12)	−0.0188 (11)
C4	0.0536 (12)	0.0651 (12)	0.0772 (15)	0.0140 (10)	0.0246 (11)	0.0023 (11)
C5	0.0737 (15)	0.0639 (13)	0.0851 (17)	−0.0010 (11)	0.0055 (13)	−0.0114 (12)
C6	0.0408 (10)	0.0650 (12)	0.0670 (13)	0.0005 (9)	0.0045 (9)	0.0064 (10)
C7	0.0422 (11)	0.0737 (13)	0.0854 (16)	0.0109 (10)	0.0174 (11)	0.0113 (12)
C8	0.0570 (12)	0.0510 (10)	0.0686 (13)	0.0036 (9)	0.0069 (10)	0.0019 (10)
C9	0.0494 (11)	0.0588 (11)	0.0575 (12)	0.0071 (9)	0.0054 (9)	0.0025 (9)
C10	0.0522 (11)	0.0548 (10)	0.0559 (11)	0.0085 (9)	0.0160 (9)	0.0002 (9)
C11	0.0619 (12)	0.0656 (12)	0.0471 (11)	−0.0049 (10)	0.0046 (9)	−0.0045 (9)
C12	0.0418 (10)	0.0519 (10)	0.0479 (10)	−0.0003 (8)	0.0098 (8)	0.0075 (8)
C13	0.0516 (11)	0.0514 (10)	0.0552 (12)	0.0021 (8)	0.0090 (9)	−0.0013 (9)
C14	0.0481 (11)	0.0514 (10)	0.0636 (13)	0.0026 (8)	0.0097 (9)	−0.0107 (9)
C15	0.0651 (13)	0.0597 (11)	0.0494 (11)	−0.0083 (10)	0.0125 (9)	−0.0011 (9)
C16	0.0381 (9)	0.0540 (10)	0.0486 (10)	0.0015 (7)	0.0126 (8)	−0.0054 (8)
C17	0.0421 (10)	0.0535 (10)	0.0444 (10)	0.0013 (8)	0.0112 (8)	−0.0024 (8)
C18	0.0456 (10)	0.0557 (10)	0.0508 (11)	−0.0116 (8)	0.0103 (8)	−0.0006 (9)
C19	0.0395 (9)	0.0494 (9)	0.0443 (10)	0.0025 (7)	0.0099 (7)	0.0055 (8)

### Geometric parameters (Å, °)

**Table:**

O1—C13	1.416 (2)	C6—C7	1.382 (3)
O1—C17	1.450 (2)	C6—C12	1.391 (3)
N1—C18	1.347 (2)	C6—H6A	0.9300
N1—C13	1.466 (2)	C7—H7A	0.9300
N1—C3	1.474 (2)	C8—C14	1.377 (3)
C1—C2	1.514 (4)	C8—C9	1.378 (3)
C1—C3	1.528 (3)	C8—H8A	0.9300
C1—H1A	0.9700	C9—C16	1.386 (3)
C1—H1B	0.9700	C9—H9A	0.9300
O2—C18	1.234 (2)	C10—C19	1.388 (3)
C2—C13	1.520 (3)	C10—H10A	0.9300
C2—H2A	0.9700	C11—C14	1.383 (3)
C2—H2B	0.9700	C11—C15	1.385 (3)
C3—H3A	0.9700	C11—H11A	0.9300
C3—H3B	0.9700	C12—C19	1.402 (3)
C4—C7	1.366 (3)	C12—C18	1.500 (3)
C4—C10	1.383 (3)	C13—H13A	0.9800
C4—H4A	0.9300	C15—C16	1.388 (3)
C5—C14	1.509 (3)	C15—H15A	0.9300
C5—H5A	0.9600	C16—C17	1.504 (2)
C5—H5B	0.9600	C17—C19	1.524 (2)
C5—H5C	0.9600	C17—H17A	0.9800
			
C13—O1—C17	114.55 (13)	C8—C9—C16	121.24 (19)
C18—N1—C13	123.97 (15)	C8—C9—H9A	119.4
C18—N1—C3	123.30 (17)	C16—C9—H9A	119.4
C13—N1—C3	112.62 (16)	C4—C10—C19	120.91 (19)
C2—C1—C3	103.64 (19)	C4—C10—H10A	119.5
C2—C1—H1A	111.0	C19—C10—H10A	119.5
C3—C1—H1A	111.0	C14—C11—C15	121.61 (19)
C2—C1—H1B	111.0	C14—C11—H11A	119.2
C3—C1—H1B	111.0	C15—C11—H11A	119.2
H1A—C1—H1B	109.0	C6—C12—C19	120.33 (18)
C1—C2—C13	104.25 (19)	C6—C12—C18	118.34 (17)
C1—C2—H2A	110.9	C19—C12—C18	121.32 (16)
C13—C2—H2A	110.9	O1—C13—N1	112.46 (15)
C1—C2—H2B	110.9	O1—C13—C2	108.58 (16)
C13—C2—H2B	110.9	N1—C13—C2	102.88 (16)
H2A—C2—H2B	108.9	O1—C13—H13A	110.9
N1—C3—C1	102.74 (16)	N1—C13—H13A	110.9
N1—C3—H3A	111.2	C2—C13—H13A	110.9
C1—C3—H3A	111.2	C8—C14—C11	117.62 (18)
N1—C3—H3B	111.2	C8—C14—C5	121.05 (19)
C1—C3—H3B	111.2	C11—C14—C5	121.30 (19)
H3A—C3—H3B	109.1	C11—C15—C16	120.43 (18)
C7—C4—C10	120.74 (19)	C11—C15—H15A	119.8
C7—C4—H4A	119.6	C16—C15—H15A	119.8
C10—C4—H4A	119.6	C9—C16—C15	117.76 (17)
C14—C5—H5A	109.5	C9—C16—C17	119.98 (17)
C14—C5—H5B	109.5	C15—C16—C17	122.24 (16)
H5A—C5—H5B	109.5	O1—C17—C16	106.89 (14)
C14—C5—H5C	109.5	O1—C17—C19	111.69 (14)
H5A—C5—H5C	109.5	C16—C17—C19	115.45 (14)
H5B—C5—H5C	109.5	O1—C17—H17A	107.5
C7—C6—C12	120.2 (2)	C16—C17—H17A	107.5
C7—C6—H6A	119.9	C19—C17—H17A	107.5
C12—C6—H6A	119.9	O2—C18—N1	123.04 (18)
C4—C7—C6	119.7 (2)	O2—C18—C12	121.86 (18)
C4—C7—H7A	120.1	N1—C18—C12	115.10 (16)
C6—C7—H7A	120.1	C10—C19—C12	118.08 (17)
C14—C8—C9	121.34 (19)	C10—C19—C17	123.07 (17)
C14—C8—H8A	119.3	C12—C19—C17	118.83 (16)
C9—C8—H8A	119.3		

### Hydrogen-bond geometry (Å, °)

**Table:**

*D*—H···*A*	*D*—H	H···*A*	*D*···*A*	*D*—H···*A*
C5—H5B···O2^i^	0.96	2.42	3.354 (3)	163.
C17—H17A···O2^ii^	0.98	2.38	3.357 (3)	174.

Symmetry codes: (i) −*x*, *y*+1/2, −*z*+1/2; (ii) −*x*, −*y*+1, −*z*.
